# Insulin-like Growth Factor Binding Protein 3 Increases Mouse Preimplantation Embryo Cleavage Rate by Activation of IGF1R and EGFR Independent of IGF1 Signalling

**DOI:** 10.3390/cells11233762

**Published:** 2022-11-24

**Authors:** Charmaine J. Green, Miriam Span, Monique H. Rayhanna, Marisa Perera, Margot L. Day

**Affiliations:** School of Medical Sciences, Faculty of Medicine and Health, University of Sydney, Camperdown, NSW 2050, Australia

**Keywords:** insulin-like growth factor binding protein 3, insulin-like growth factor 1, preimplantation embryo, mice

## Abstract

The viability of embryos cultured *in vitro* is poor compared to those that develop *in vivo*. The lack of maternally derived growth factors *in vitro* may contribute to this problem. Insulin-like growth factor binding protein 3 (IGFBP3) is one such growth factor that has been identified in the maternal reproductive system. This study examined the role of autocrine and exogenous IGFBP3 in mouse preimplantation embryos. Embryos expressed IGFBP3 across all stages of preimplantation development, and addition of exogenous IGFBP3 to embryo culture media increased the rate of development to the 2-, 4-, 5-, and 8-cell stages. Addition of inhibitors of the IGF1 and EGF receptors prevented this IGFBP3-mediated improvement in developmental rate, but the effect was not cumulative, indicating that both receptors are transactivated downstream of IGFBP3 as part of the same signalling pathway. Acute exposure to IGFBP3 increased phosphorylation of Akt and rps6 in 4–8 cell embryos, suggesting activation of the PI3-kinase/Akt pathway downstream of the IGF1 and EGFR receptors to promote cell proliferation and survival. In conclusion, addition of IGFBP3 to embryo culture media increases early cleavage rates independent of IGF1 signalling and therefore, IGFBP3 addition to IVF culture media should be considered.

## 1. Introduction

*In vivo* development of the mammalian preimplantation embryo occurs in close proximity to the maternal reproductive tract. In addition to supplying nutrition for the early embryo, the maternal reproductive tract influences embryo development by the secretion of growth factors, cytokines and amino acids which are known to affect embryo development. The preimplantation embryo itself also produces autocrine factors which improve *in vitro* embryo development in the absence of maternal factors. Insulin-like growth factor 1 (IGF1) is an important autocrine growth factor for mouse preimplantation development *in vitro* and acts to increase development to the blastocyst stage and decrease apoptosis [1].

In other systems, the IGF binding proteins (IGFBPs) regulate the action of IGF1, possibly by prolonging the half-life of IGF1 and by increasing or preventing IGF1 binding to its receptor. IGFBPs also have signalling roles that are independent to IGF1 action [2]. Thus, in addition to the direct effects of IGF1 during preimplantation embryo development, the IGFBPs may also play a critical role that has not yet been investigated. There are six members of the IGF binding protein family, which share several structural and functional characteristics including the ability to bind to IGF1 [3].

IGFBP3 protein is present at the maternal–foetal interface in many mammalian species including the human and mouse [4,5]. *IGFBP3* mRNA is expressed by the cells of the mouse reproductive tract including the oviduct, uterus and cumulus cells [4]. Additionally, IGFBP3 mRNA and protein are expressed by preimplantation embryos and, in the mouse, *IGFBP3* transcript expression is initiated at the 2–4 cell stage [6].

Studies have shown that bovine embryos developing *in vitro* express significantly reduced *IGFBP3* mRNA compared to their *in vivo* counterparts [7]. A loss of maternal and autocrine IGFBP3 during *in vitro* culture may negatively impact on development as cultured embryos have a slower cell division and undergo greater developmental arrest compared to those embryos that develop *in vivo*. Secretions by endometrial cells enhance embryo development [8] possibly due to the presence of several IGFBPs, including IGFBP3 [9]. Co-culture of mouse embryos with human oviductal cells increases production of IGFBP3 by the oviductal cells and this correlates with improved embryo development [5]. Another study conducted on women undergoing IVF treatment showed that higher concentrations of IGFBP3 in human follicular fluid, at the time of oocyte retrieval were correlated with improved early embryo development [10].

IGFBP3 may also play a role in implantation, decidualization and trophoblast invasion. In rats, IGFBP3 transcripts were detected at the decidual boundary and in the blood vessels of the endometrium following implantation [11]. In human endometrial tissue, IGFBP3 is absent during the proliferative phase and increases during the secretory phase, which marks the window for embryo implantation [12]. More recently, BMP2-mediated IGFBP3 upregulation was found to have a number of roles in the processes surrounding embryo implantation [12,13]. In *in vitro* cell models, the BMP2-ID1-IGFBP3 signalling pathway promotes human trophoblast cell invasion, as well as endothelial-like tube formation, matrix metalloprotease expression and cell migration in human endometrial stromal cells [12,13].

It is now emerging that IGFBP3 has signalling effects that are independent of IGF1 that lead to cell proliferation and survival in many cell types [14]. However, the mechanisms that underlie IGFBP3 actions are not well understood and there is no consensus on the identity of the cell-surface receptor for IGFBP3. IGFBP3 can interact with receptors for other growth factors at the cell surface, including TGFβ-R, which results in activation of the Smad signalling pathway and regulation of cell proliferation [15,16]. In addition to acting at the cell surface, IGFBP3 can be taken up into the cytoplasm and nucleus of cells. IGFBP3 has been shown to interact with nuclear receptors, mitochondrial targets, and enhance the stimulatory effect of growth factors and activate MAPK and PI3K pathways [17,18,19]. IGFBP3 can associate with Importin β, which facilitates transport of IGFBP3 into the nucleus [20]. Within the nucleus IGFBP3 interacts with nuclear hormone receptors, including the retinoic X receptor α (RXR-α) [18,19] and retinoic acid receptor α (RAR-α) [21] to regulate transcription. RXR-α has a number of binding partners through which an IGFBP3-mediated effect can occur, including Nur77, which activates apoptosis [22], and the vitamin D and thyroid hormone receptors, which inhibits transcription of genes normally responsive to these hormones [23,24]. In breast cancer cells, IGFBP3 promotes survival through a number of mechanisms, including 78-kDa glucose-regulated protein (GRP78)-mediated autophagy [25], promotion of DNA repair through formation of a nuclear complex with the epithelial growth factor receptor (EGFR) and the catalytic subunit of DNA-dependent protein kinase (DNA-PKcs) [26], and through transactivation of the EGFR via the sphingosine-1-phosphate (S1P) pathway [27].

The aim of the present study was to examine the role of IGFPB3 in preimplantation mouse embryo development, and to investigate the signalling pathways activated by IGFBP3 and the complex signalling interactions between IGFBP3 and the IGF1R and EGF receptors, which may be present in the embryo. We hypothesised that IGFBP3 will improve early embryo development and may have effects independent of its ability to interact with IGF1.

## 2. Materials and Methods

### 2.1. Media and Chemical Preparation for Embryo Collection and Culture

The medium used for embryo collection was HEPES (*N*-2-Hydroxyethylpiperazine-*N*’-2-ethanesulfonic acid) buffered modified synthetic human tubal fluid medium (HEPES mod-HTF; ~270–300 mOsm/kg, pH 7.4) containing 0.3 mg/mL bovine serum albumin (BSA; Sigma-Aldrich; St Louis, MO, USA). The medium used for embryo culture was potassium simplex optimized medium (KSOM; ~260 mOsm/kg, pH 7.4) containing 0.3 mg/mL BSA. All media were made up from stock solutions prepared from tissue culture grade reagents as previously described [28]. All reagents were purchased from Sigma Aldrich unless otherwise specified.

Recombinant mouse IGFBP3 (R&D Systems, Minneapolis, MN, USA) was reconstituted at a concentration of 100 μg/mL in sterile PBS. Human/mouse IGF1R neutralising antibody (IGF1R nAb; AF-305-NA; R&D Systems) was reconstituted at 0.2 mg/mL in PBS. Aliquots were prepared and stored at −80 °C. Erlotinib hydrochloride (Santa Cruz, Dallas, TX, USA) was reconstituted at a concentration of 5 mM in dimethyl sulfoxide (DMSO). Aliquots were prepared and stored at −20 °C.

### 2.2. Embryo Collection and Culture

Embryos were isolated from Quackenbush Swiss (QS). Procedures involving the use of animals were conducted in accordance with the Australian Code of Practice for Use of Animals in Research and were approved by the University of Sydney Animal Care and Ethics Committee (protocols 4838, 5583, 824). Mice were housed under a 12 h light: 12 h dark cycle (light; 06:00–18:00), with free access to food and water. Female mice (4–10 weeks old) were superovulated by intraperitoneal injection of 10 International Units (I.U.) of pregnant mare serum gonadotrophin (PMSG; Intervet, Sydney, Australia) followed 48 h later by intraperitoneal injection of 10 I.U. of human chorionic gonadotrophin (hCG; Intervet). Superovulated female mice were then paired with a stud male QS mouse (10–30 weeks old) overnight and checked for the presence of a vaginal plug the following day, indicating successful mating (day 1 of pregnancy). Female mice were euthanized 20–22 or 60–65 h post hCG administration in order to isolate zygote or 4–8 cell stage embryos respectively. Zygotes were isolated from the oviducts into HEPES mod-HTF (37 °C) and treated with hyaluronidase (1 mg/mL in HEPES-modHTF) (Sigma-Aldrich, St. Louis, MO, USA) to remove cumulus cells. Zygotes were then collected and washed at least three times in KSOM. To isolate fresh 4–8 cell embryos, the oviducts were flushed with HEPES mod-HTF.

Embryos were cultured from the zygote stage at low density (1 embryo/100 µL) in round bottom 96-well plates (Corning, NY, USA) containing KSOM, pre-warmed to 37 °C and equilibrated at 5% CO_2_ for a minimum of two hours. To calculate the time spent by embryos in each cell stage before their next division, zygotes were placed in the IncuCyteZOOM Live Cell Imaging incubator (Essen Biosciences, Ann Arbor, MI, USA) in 96-well culture plates. Hourly photographs were taken in each well of the plate, thus allowing embryo development to be tracked in greater detail. Embryos were scored according to their developmental stage (3-cell, 4-cell, 5-cell, 8-cell, compacted, morula, cavitated and blastocyst) per hour. Blastocyst development and hatching from the zona pellucida was noted on day six of development (144 h post-hCG). The medium was not changed over the six-day period.

### 2.3. Western Blotting

Embryo protein preparation and Western blotting was performed as described previously [1]. Membranes were probed with primary antibodies using 1:500 rabbit anti-phospho-Akt (Ser473) (Cell Signalling, Danvers, MA, USA, #4058), 1:100 p-RPS6 (Ser235/236) (Cell Signalling #4856S) or 1:1000 mouse anti-α-tubulin (Sigma-Aldrich, #T9026). Membranes were washed in Tris-buffered saline + Tween 20 (TBST; 10 mM Tris–HCl, pH 7.6, 150 mM NaCl and 0.1% Tween 20) and subsequently incubated for 2 h with either 1:4000 Donkey anti-Rabbit IRDye 800CWLI-COR Biosciences, Lincoln, NE, USA) or 1:4000 Donkey anti-Mouse IRDye 680LT (LI-COR Biosciences). Proteins were visualized using the Odyssey infrared imager and Odyssey application software, version 3 (LI-COR Biosciences). Densitometry was performed using Image-J software v1.53c (National Institute of Health, Bethesda, MD, USA).

### 2.4. Immunofluorescent Staining of Oocytes and Embryos

Immunofluorescent staining was performed as described previously [1]. Briefly, embryos were fixed in 4% PFA for 15 min and permeabilized with PBS + PVA + 0.3% Triton X 100 for 30 min. Blocking was carried out in PBS + PVA + 0.1% Tween-20 + 0.7% BSA (PPTB) for 30 min. Primary and secondary antibodies were diluted in PPTB. Samples were incubated with primary antibody (rabbit anti-IGFBP3; Santa Cruz #SC-902) overnight at 4 °C and secondary antibody (anti-rabbit Alexa 488; Life Technologies, Carlsbad, CA, USA) for two hours at room temperature. Isotype controls were performed, in which cells were incubated with purified rabbit immunoglobulin (IgG; Sigma) in place of the primary antibody. Fluorescence was visualized using the LSM 510 Meta confocal microscope (Carl Zeiss, Oberkochen, Germany) using 405 nm and 488 nm lasers at 40 X objective. Images were analyzed using LSM Image Browser software (Carl Zeiss).

### 2.5. Statistical Analysis

Embryos were randomly allocated into control and treatment groups. Chi-squared analysis of the effects of culture conditions on blastocyst development and hatching were performed on at least three pooled experiments unless stated otherwise and compared the control group to the treatment group. Results comparing lengths of cell cycle between control and IGFBP3 treated embryos were compared by unpaired *t*-test (performed using GraphPad Prism v9) and were expressed as the mean ± standard error of the mean (SEM). Results comparing lengths of cell cycle between multiple treatment groups were compared by one-way ANOVA followed by Tukey’s post-hoc test and were expressed as the mean ± standard error of the mean (SEM). Results comparing average number of cells in blastocysts between treatment groups were compared by unpaired *t*-test. Western blot analysis was performed on groups of 150 embryos (n = 3). Densitometry was performed using Image-J software. The optical density of pAkt and pRps6 bands were normalised to α-tubulin and expressed relative to the control as the mean ± standard error of the mean (SEM), followed by statistical analysis using unpaired *t*-tests.

## 3. Results

### 3.1. IGFBP3 Is Expressed across All Stages of Mouse Preimplantation Embryo Development

**Figure 1:**
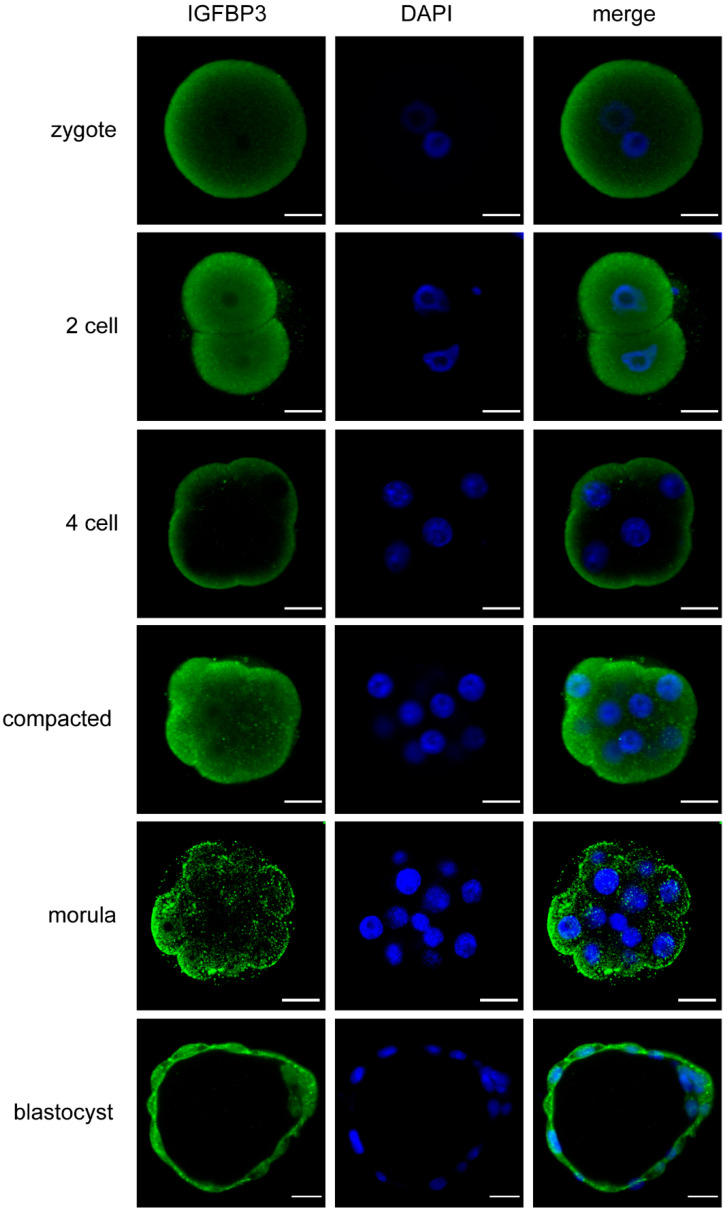
IGFBP3 expression in preimplantation embryos. Representative confocal images of preimplantation stage embryos isolated from the reproductive tract and immunostained for IGFBP3 (green) and co-stained with DAPI (blue). Scale bar represents 20 μm. Images are representative of at least 10 embryos per stage.

IGFBP3 mRNA is known to be expressed by preimplantation embryos [7]; however, protein expression and localisation has not yet been determined. Immunofluorescent staining revealed that IGFBP3 was localized in the cytoplasm, particularly the cortical cytoplasm and membrane, during all stages of mouse preimplantation development from the zygote stage onwards (Figure 1). From the 4–8-cell stage, embryos appeared to have a more distinct apical localisation of IGFBP3. In blastocyst stage embryos IGFBP3 was located in the cytoplasm, nucleus, cell–cell junctions and on the basal and apical surface of the cells in the trophectoderm. The inner cell mass (ICM) also expressed IGFBP3.

### 3.2. IGFBP3 Increases the Developmental Rate of Preimplantation Embryos In Vitro

**Figure 2:**
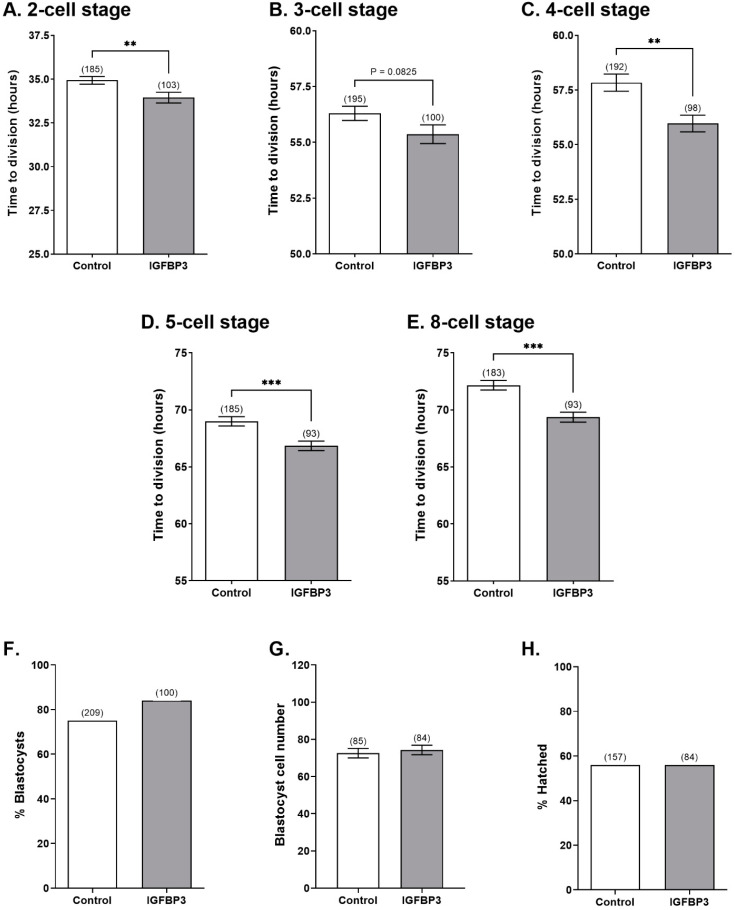
IGFBP3 decreases time to progress through the cell cycle. Mouse zygotes were isolated and cultured at a density of 1 embryo/100 μL in the presence or absence of 100 ng/mL IGFBP3. Rate of development was measured using mean time taken, in hours post hCG injection, for the embryo to divide to the (**A**) 2-cell, (**B**) 3-cell, (**C**) 4-cell, (**D**) 5-cell, and (**E**) 8-cell stages ± SEM. Embryos that failed to develop to the blastocyst stage were excluded. Statistical analysis was performed by unpaired *t*-test. On day 6 of development, the percentage of embryos that developed to the blastocyst stage was recorded (**F**), and blastocysts were fixed and stained with DAPI to count the number of cells within each blastocyst (**G**). The percentage of blastocysts that hatched from the zona pellucida (**H**) was also recorded. The percentage of embryos that reached the blastocyst and hatched blastocyst stages in control and IGFBP3 treatments were compared using a chi-squared test. Average cell numbers between control and IGFBP3 treatments were compared using an unpaired *t*-test. Data shown represent 6–11 independent experiments (number of embryos are in parentheses). ** = *p <* 0.01, *** = *p <* 0.001.

To identify the effects of IGFBP3 on early cell division, zygotes were cultured in the presence or absence of IGFBP3, and the developmental stages were tracked hourly using the IncuCyte ZOOM time lapse imaging system. IGFBP3 increased the rate of embryo development to the 2-cell, 4-cell, 5-cell, and 8-cell stages compared to embryos cultured in KSOM alone (Figure 2A–E). Treatment with IGFBP3 had no effect on the percentage of embryos that developed to the blastocyst stage, cell numbers in the blastocysts or the percentage of blastocysts that were hatching (Figure 2F–H). 

### 3.3. The IGF1 and EGF Receptors Are Required for Stimulatory IGFBP3 Signalling in the Preimplantation Embryo

The mechanisms by which IGFBP3 exerts its proliferative effects are largely unknown, and are thought to be independent of IGF1. In epithelial cells, IGFBP3 exerts its effects via the transactivation of the IGF1R and EGFR [29]. To investigate possible transactivation of IGF1R and EGFR by IGFBP3 in embryos, the effect of blocking the IGF1R and EGFR, on the stimulatory effects of IGFBP3 on development was investigated by use of Erlotinib and an IGF1R neutralising antibody (IGF1RnAb), respectively. The EGFR inhibitor Erlotinib acts by competing with the ATP binding site of the EGFR to inhibit phosphorylation of the receptor. The IGFR1nAb binds to the extracellular domain of the IGF1R to prevent IGF1 binding and also triggers internalization of the receptor, thereby preventing autophosphorylation and activation of the receptor [30]. The addition of IGF1nAb to embryo culture reduces development to the blastocyst stage, hatching, and total cell numbers in the blastocyst [31].

**Figure 3:**
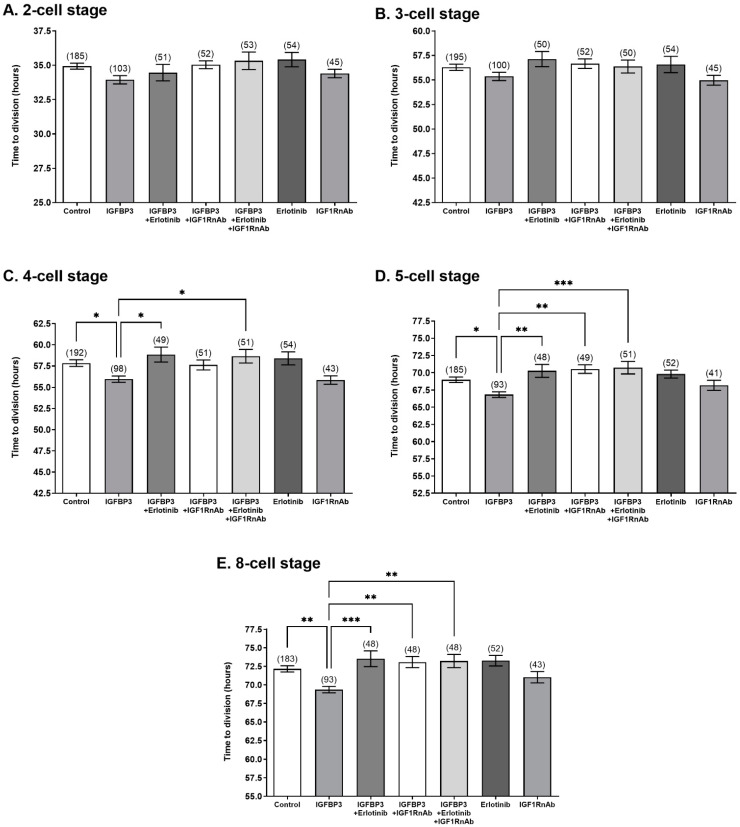
IGF1R and EGFR inhibitors prevent the IGFBP3-mediated increase in developmental rate to the 4- to 8-cell stages. Mouse zygotes were isolated and cultured at a density of 1 embryo/100 μL in medium alone (control) or medium containing 100 ng/mL IGFBP3, 100 ng/mL IGFBP3 plus 2 μM Erlotinib, 100 ng/mL IGFBP3 plus 2 mg/mL IGF1RnAb, 100 ng/mL IGFBP3 plus 2 μM Erlotinib plus 2 mg/mL IGF1RnAb, 2 μM Erlotinib, or 2 mg/mL IGF1RnAb. Rate of development was measured using mean time taken, in hours post hCG injection, for the embryo to divide to the (**A**) 2-cell, (**B**) 3-cell, (**C**) 4-cell, (**D**) 5-cell, (**E**) 8-cell stages ± SEM. Embryos that failed to develop to the blastocyst stage were excluded. Statistical analysis was performed by one-way ANOVA with Tukey’s post hoc test. Data shown represent 6–9 independent experiments (number of embryos are in parentheses). * *p <* 0.05, ** *p <* 0.01, *** *p <* 0.001.

**Figure 4:**
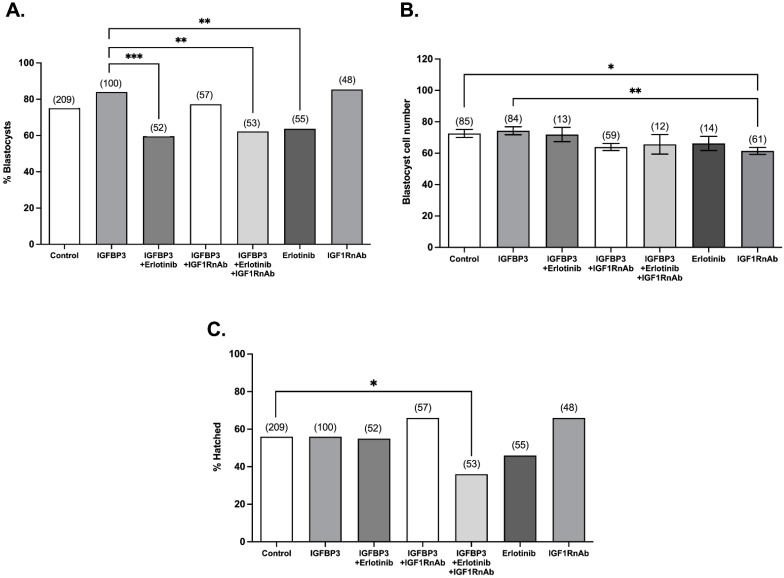
EGFR inhibition decreases the percentage of embryos that reach the blastocyst stage. Mouse zygotes were isolated and cultured at a density of 1 embryo/100 μL in medium alone (control) or medium containing 100 ng/mL IGFBP3, 100 ng/mL IGFBP3 plus 2 μM Erlotinib, 100 ng/mL IGFBP3 plus 2 mg/mL IGF1RnAb, 100 ng/mL IGFBP3 plus 2 μM Erlotinib plus 2 mg/mL IGF1RnAb, 2 μM Erlotinib, or 2 mg/mL IGF1RnAb. On day 6 of culture, the percentage of embryos that developed to the blastocyst stage (**A**) was recorded, and blastocysts were fixed and stained with DAPI to count the number of cells within each blastocyst (**B**). The percentage of blastocysts that hatched from the zona pellucida (**C**) was also recorded. The percentage of embryos that reach the blastocyst and hatched blastocyst stages between treatments were compared using a chi-squared test. The average cell number between treatments was compared using one-way ANOVA with Tukey’s post hoc test. Data shown represent 6–9 independent experiments (number of embryos are in parentheses). * *p <* 0.05, ** *p <* 0.01, *** *p <* 0.001.

In the present study, the presence of either the IGF1RnAb or Erlotinib prevented the IGFBP3-induced decrease in the time to division to the 5- and 8-cell stages (Figure 3). Erlotinib, but not IGF1RnAb, also prevented the IGFBP3-induced decrease in the time to division to the 4-cell stage (Figure 3). Culture of embryos in the presence of Erlotinib or IGF1RnAb alone had no effect on cell division rate compared to the control (Figure 3). The effect of IGFBP3 at the 4-, 5-, and 8-cell stages was also abolished when embryos were cultured in the presence of IGFBP3 with Erlotinib and IGF1R-nAb together; however, no cumulative effect between Erlotinib and IGF1RnAb was observed. Culture of embryos in the presence of Erlotinib or IGFBP3 and Erlotinib with or without IGF1RnAb decreased the percentage of embryos that developed to the blastocyst stage compared to IGFBP3 alone (Figure 4A). Culture of embryos in the presence of IGF1RnAb decreased the number of cells within each blastocyst compared to control embryos (Figure 4B).

### 3.4. Short Term IGFBP3 Treatment Increases Phosphorylation of Akt and rps6

**Figure 5:**
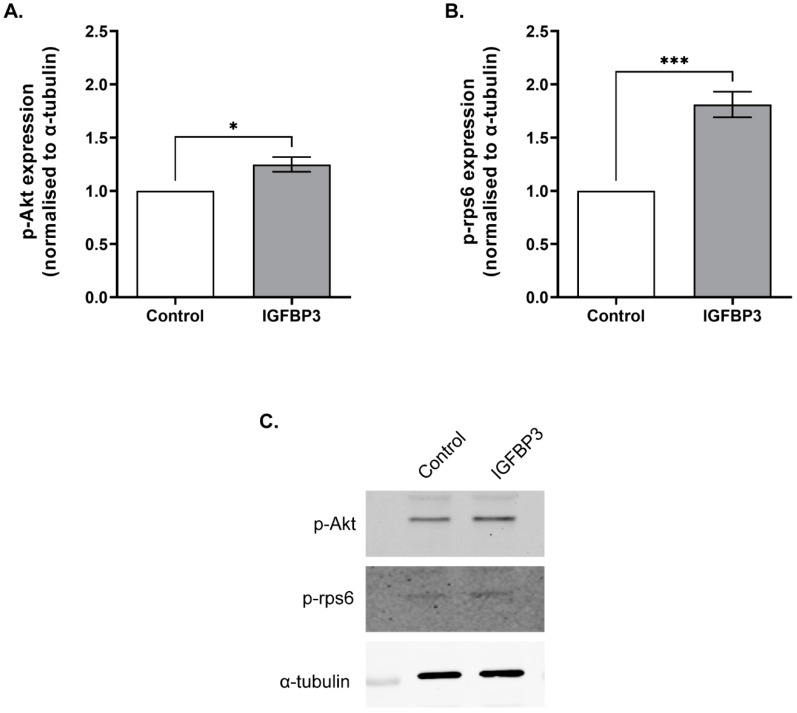
IGFBP3 increases phosphorylation of rpS6 and Akt. Effect of 100 ng/mL IGFBP3 on (**A**) phospho-Akt levels and (**B**) phospho-rps6 in 4–8 cell embryos, detected by Western blotting. Data are average phosphorylated protein band intensities (±SEM, n = 3) normalised to α-tubulin and are expressed relative to the control. (**C**) Representative Western blot of 4–8 cell embryos exposed to 100 ng/mL IGFBP3 for 10 min. Western blots were probed for anti-p-Akt (upper panel) anti-p-rps6 (middle panel) and anti-α-tubulin (lower panel). Statistical analysis was performed using an unpaired *t*-test. * = *p <* 0.05, *** = *p <* 0.001.

Previous studies in epithelial cells where IGFBP3 had been knocked down showed significantly reduced phosphorylation of Ribosomal Protein S6 (rpS6) and Akt following stimulation of the IGF1R [32]. To investigate the effect of short-term exposure to IGFBP3 on the activation of the PI3 kinase/Akt and S6-kinase/rpS6 signalling pathways in preimplantation embryos, 4–8-cell embryos were isolated from the reproductive tract and cultured in KSOM for 30 min. Embryos were then treated with IGFBP3 for 10 min and assessed for phosphorylation of Akt or rpS6 by Western blotting. An increase in phosphorylation of rpS6 and Akt was observed following IGFBP3 treatment compared to control embryos (Figure 5).

## 4. Discussion

Culture of preimplantation embryos can result in the dilution of autocrine factors. The absence of growth factors in embryo culture media may cause imbalances and stress to the embryos [33] and slow embryo development [34,35]. Recently, IGFBP3 has been implicated to have a caretaker role in the cellular stress response including the promotion of DNA repair, induction of autophagy, and regulation of pro-survival signalling pathways [36,37]. Together, this implies that IGFBP3 may be an important molecule to include in the culture environment of preimplantation embryos.

*IGFBP3* mRNA is expressed in preimplantation embryos; however, expression is decreased in cultured embryos [7]. In the present study, IGFBP3 protein was shown to be present throughout the preimplantation period. IGFBP3 was increased apically from the 4–8 cell stage, suggesting the importance of IGFBP3 at this period. Additionally, IGFBP3 was strongly expressed at the blastocyst stage, particularly in the trophectoderm. This supports recent literature implicating the involvement of IGFBP3 in blastocyst implantation and trophoblast cell invasion [11,12,13].

In the present study, addition of exogenous IGFBP3 during preimplantation embryo development resulted in an acceleration of development to the 2-, 4-, 5-, and 8-cell stages. To our knowledge this is the first study demonstrating the effect of addition of IGFBP3 to the culture media of mouse preimplantation embryos. These findings support previous studies which inferred the benefit of IGFBP3 on embryo development as it is present in the secretions of endometrial and oviductal cells [5,8,9].

The addition of IGFBP3 to the culture media of preimplantation embryos may increase the rate of cell division closer to that observed *in vivo* by activating intracellular signalling pathways. IGFBP3 is known to interact with several proteins, including those in serum, extracellular matrix, and the cytoplasm and nucleus of cells [37]. IGFBP3 is able to enter the cell through a number of mechanisms, including interaction with the plasma membrane via glycosaminoglycan- and heparin-binding domains [38,39,40], and clathrin-mediated endocytosis of transferrin-bound IGFBP3 [41]. Cytoplasmic IGFBP3 can translocate to the nucleus due to the presence of a nuclear localisation signal in its C-terminus, enabling binding to nuclear transport protein importin-β [42]. Once in the nucleus, IGFBP3 is involved in regulation of gene transcription and DNA repair through interactions with nuclear binding partners such as retinoid X receptor-α, EGFR, and histone 3 [26,43,44,45]. IGFBP3 may also exert its effects by direct interaction with receptors in the plasma membrane. Several receptors have been identified to bind with IGFBP3, including TGF-β receptors I, II, and V [46], and the novel death receptor IGFBP3R [47]. Activation of these receptors may lead to activation of signalling pathways in the embryo that promote cell proliferation and survival.

Two notable targets downstream of IGFBP3 signalling in other cell types are the IGF1R and EGFR [26,29,36]. In the present study, culture of embryos with IGFBP3 in the presence of inhibitors of EGFR and IGF1R prevented the increase in developmental rate seen at the 4-, 5-, and 8-cell stages. This effect was not cumulative when both inhibitors were present in culture, suggesting that both the EGFR and IGF1R undergo IGFBP3-mediated transactivation as part of the same signalling pathway.

A major signalling pathway downstream of the EGFR and IGF1R is the PI3-kinase (P13K)/Akt pathway [48]. Activation of the PI3K/Akt pathway is associated with cell survival and proliferation, and results in phosphorylation of rps6, a major ribosomal protein [49,50]. In embryos, inhibition of Akt during *in vitro* oocyte maturation reduces maturation rate, cumulus cell expansion, cleavage and blastocyst development [51]. Additionally, deletion of a *rps6* allele in oocytes to produce heterozygous embryos results in embryonic lethality at E5.5 due to cell cycle arrest and apoptosis [52]. Together, this highlights the necessity of PI3K/Akt/rps6 signalling in normal preimplantation embryo development. In our study, treatment of embryos with IGFBP3 increased phosphorylation of Akt and rps6. This is consistent with previous literature, in which IGFBP3 activated the rps6 pathway in epithelial cells [32], and activated Akt via the IGF1R in umbilical vein endothelial cells [53]. However, in hepatic stellate cells IGFBP3 induced cell proliferation and migration through integrin β1-mediated Akt activation independently of the IGF1R [54], and so the mechanism of Akt activation in the embryo should be investigated.

IGFBP3-mediated activation of the EGFR and IGF1R has been suggested previously in breast cancer epithelial cells, in which treatment of cells with IGFBP3 resulted in ligand-stimulated EGFR and IGF1R activation and subsequent downstream DNA synthesis [29]. This study found that activation of sphingosine kinase 1 (SphK1) was necessary for IGFBP3-mediated transactivation of the IGF1R and EGFR [29]. Sphk1 phosphorylates the lipid sphingosine to produce sphingosine-1-phosphate (S1P). S1P is a signalling lipid which can be translocated extracellularly to act as a ligand for a family of G-protein coupled receptors (S1PRs), and thereby activates intracellular signalling pathways, leading to cell proliferation and survival [55]. In epithelial cell types, treatment with exogenous IGFBP3 increases Sphk1 gene expression and phosphorylation of Sphk1, which leads to the production of S1P [29,53,56]. Inhibition of S1P production inhibits the effects of IGFBP3 on intracellular signalling [29]. Both IGFBP3 and S1P-S1PR signalling have been implicated in transactivation of receptors including IGF1R and EGFR [29,56,57]. Mouse embryonic fibroblasts treated with S1P show increased phosphorylation of EGF receptors and activation of the Ras/mitogen activated protein kinase (MAPK) pathway [56]. Therefore, the S1P signalling pathway should be investigated as a potential intermediate in the IGFBP3/EGFR/IGF1R signalling pathway in the embryo.

In summary, the addition of exogenous IGFBP3 to preimplantation embryo culture medium increases developmental rate during early embryo cell division. It appears that IGFBP3 requires the EGFR and IGF1R to mediate its effects in the embryo. However, the mechanism by which the EGFR and IGF1R are involved in IGFBP3 signalling remains to be understood. In other cell types, IGFBP3 can activate the EGFR and IGF1R via transaction involving the S1P signalling pathway [53], and this pathway warrants investigation in the embryo. In the present study, IGFBP3 activates a number of signalling molecules including Akt and rps6. These pathways are important in cell survival and the progression of cells through the cell cycle. As embryo development is delayed *in vitro*, addition of IGFBP3 to the culture medium of preimplantation embryos may be beneficial to the developmental outcome of cultured embryos.

## Data Availability

All data is included in the published article. Data and materials will be made available upon request.
